# Supplementary figures and images for: Genetically distinct within-host subpopulations of hepatitis C virus persist after Direct-Acting Antiviral treatment failure
Source: PLoS Pathog. 2025 Apr 1;21(4):e1012959. doi: 10.1371/journal.ppat.1012959 (PMC11981120; doi:10.1371/journal.ppat.1012959)

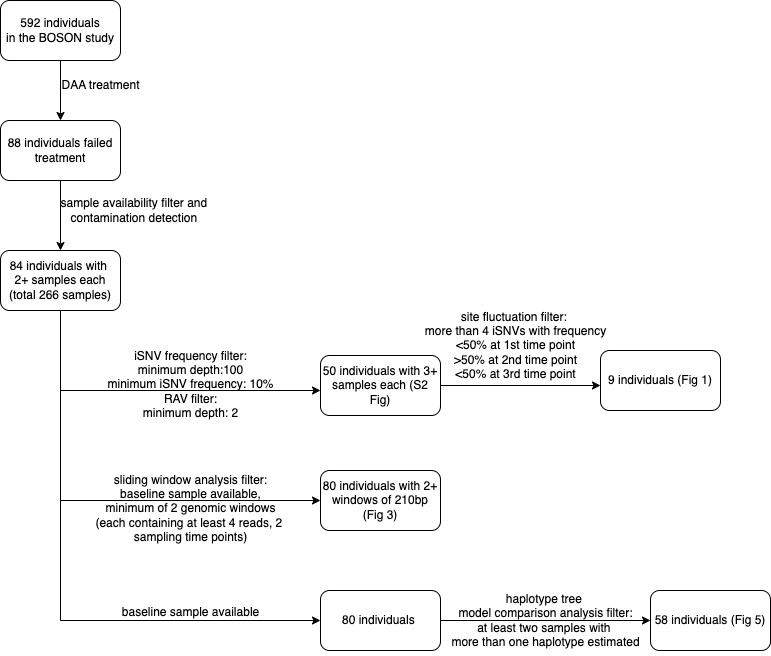

Supplement: S1 Fig — (DOCX) [file ppat.1012959.s001.docx]

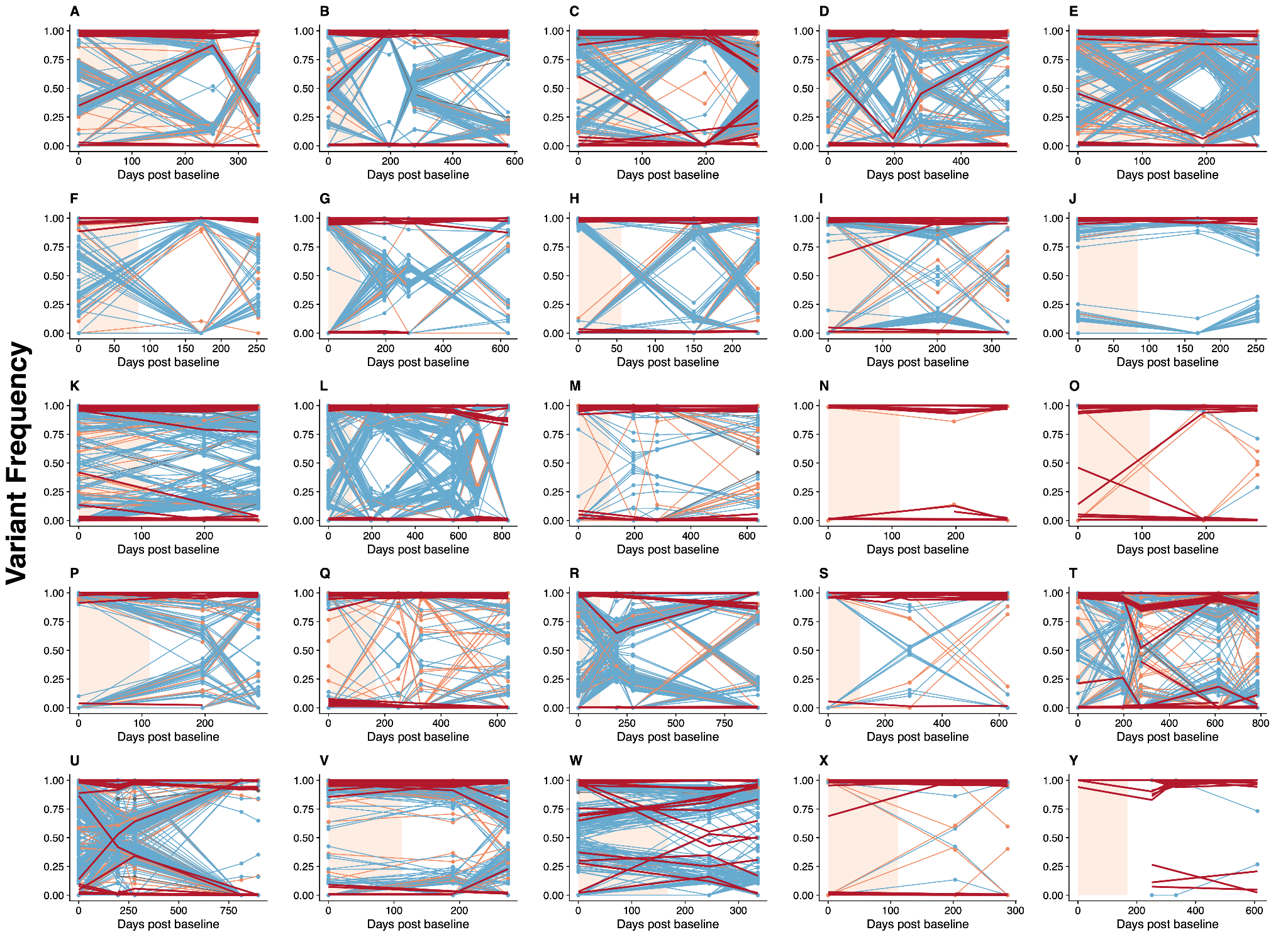


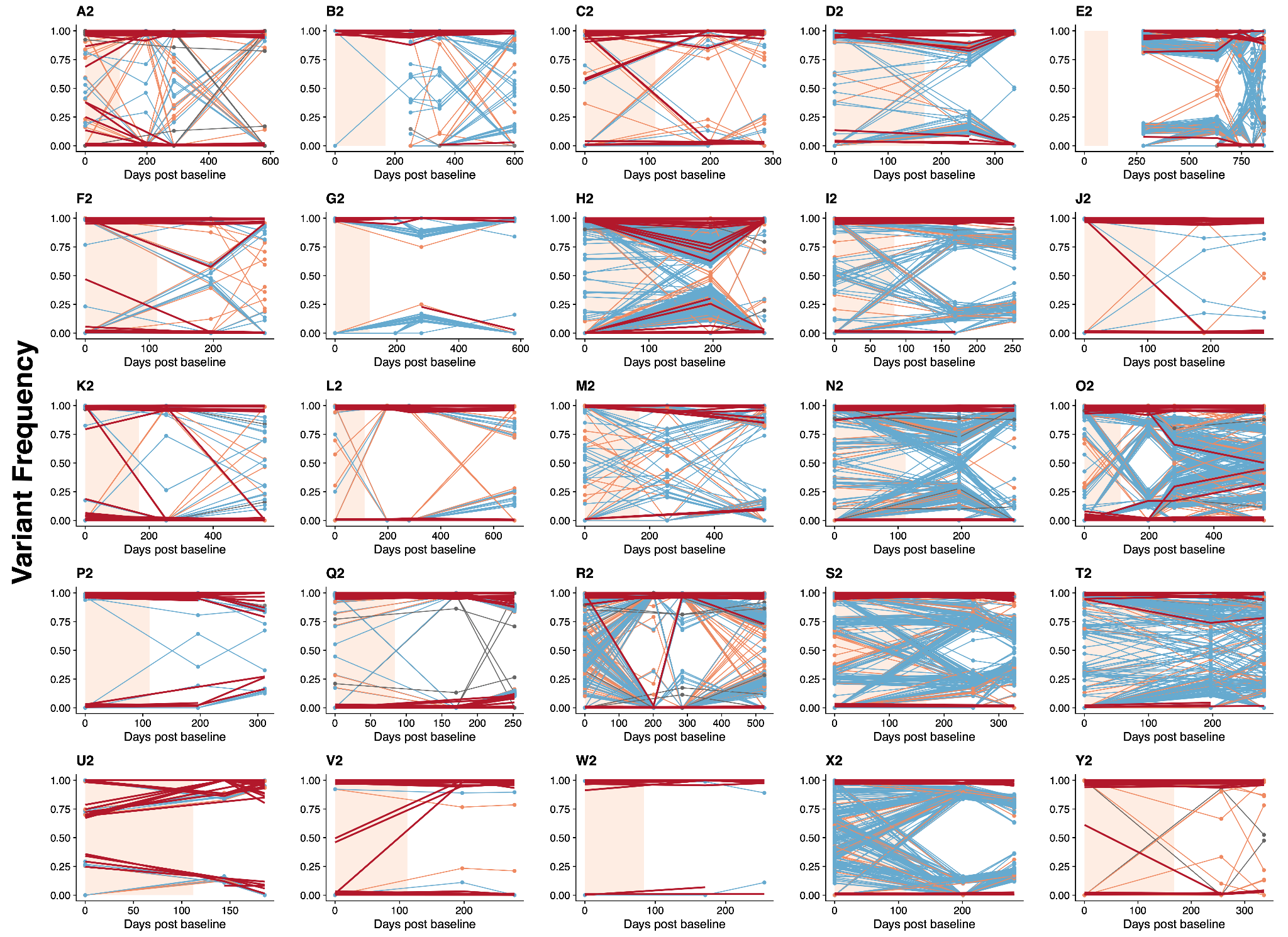

Supplement: S2 Fig — All nucleotide variants with a frequency above 10% in at least one sample were traced across all sampling time points, with synonymous changes in blue, nonsynonymous changes in orange and non-coding in grey. All relevant resistance-associated variants (RAV) trajectories are in red. The peach-coloured shade indicates the duration of the DAA treatment. All variants that became fixed (frequency increased to and remained at above 90%) or were purged (frequency decreased to and remained at below 10%) after the first sampling time point were not included. Patient IDs were assigned randomly, for example, there is no relevance between patient A and patient A2. (DOCX) [file ppat.1012959.s002.docx]

**
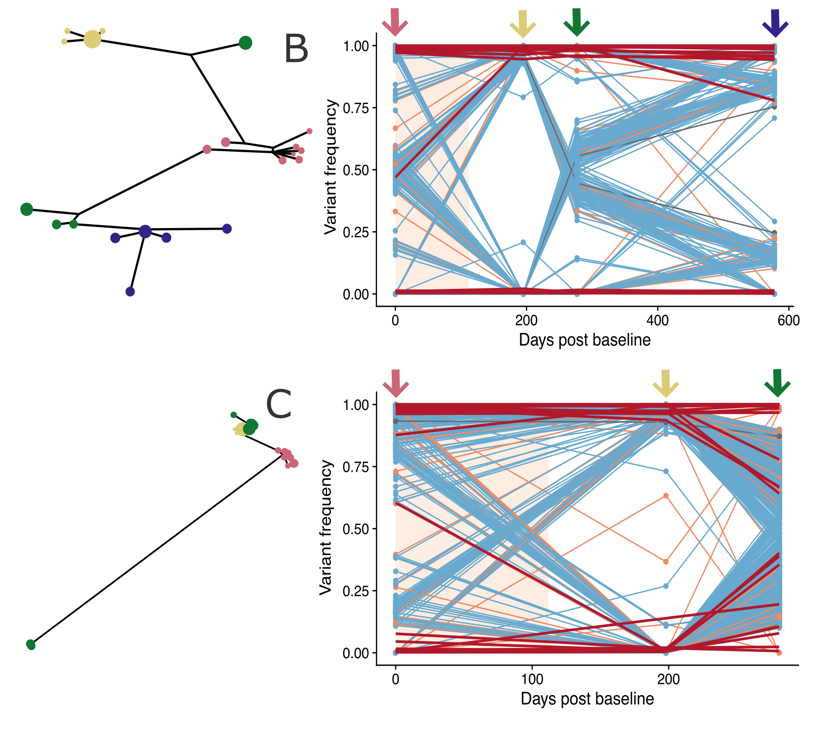
**

Supplement: S3 Fig — All iSNVs with a frequency above 10% in at least one sample were traced across all sampling time points, with synonymous changes in blue, nonsynonymous changes in orange and non-coding in grey. All resistance-associated variants (RAV) trajectories are in red. The peach-coloured shade indicates the duration of the DAA treatment. The coloured arrows on top of the trajectories and the coloured tips in the haplotype phylogenies represent the different sampling time points (light red: baseline; yellow: PT12 (12 weeks post treatment); green: PT24 (24 weeks post treatment); purple: BRT (baseline before re-treatment)). (DOCX) [file ppat.1012959.s003.docx]

**
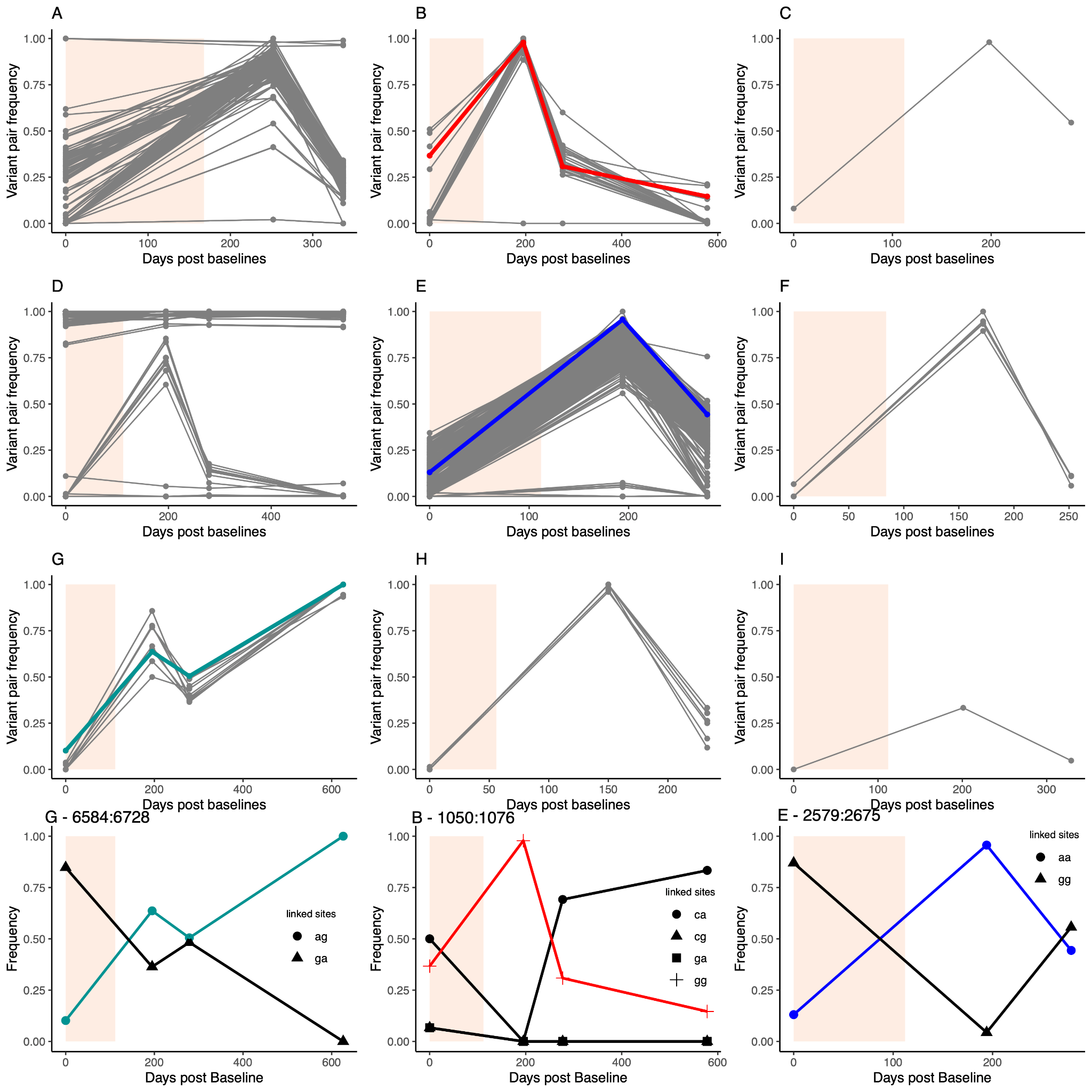
**

Supplement: S4 Fig — Panel G - 6584:6728 shows the trajectories of paired variants (day 0 frequency > 5%) between genomic position 6584 and 6728 for Patient G. Panel B - 1050:1076 shows the trajectories of all paired variants (day 0 frequency > 5%) between genomic position 1050 and 1076 for Patient B. Panel E - 2579:2675 shows the trajectories of paired variants (day 0 frequency > 5%) between genomic position 2579 and 2675 for Patient E. (DOCX) [file ppat.1012959.s004.docx]

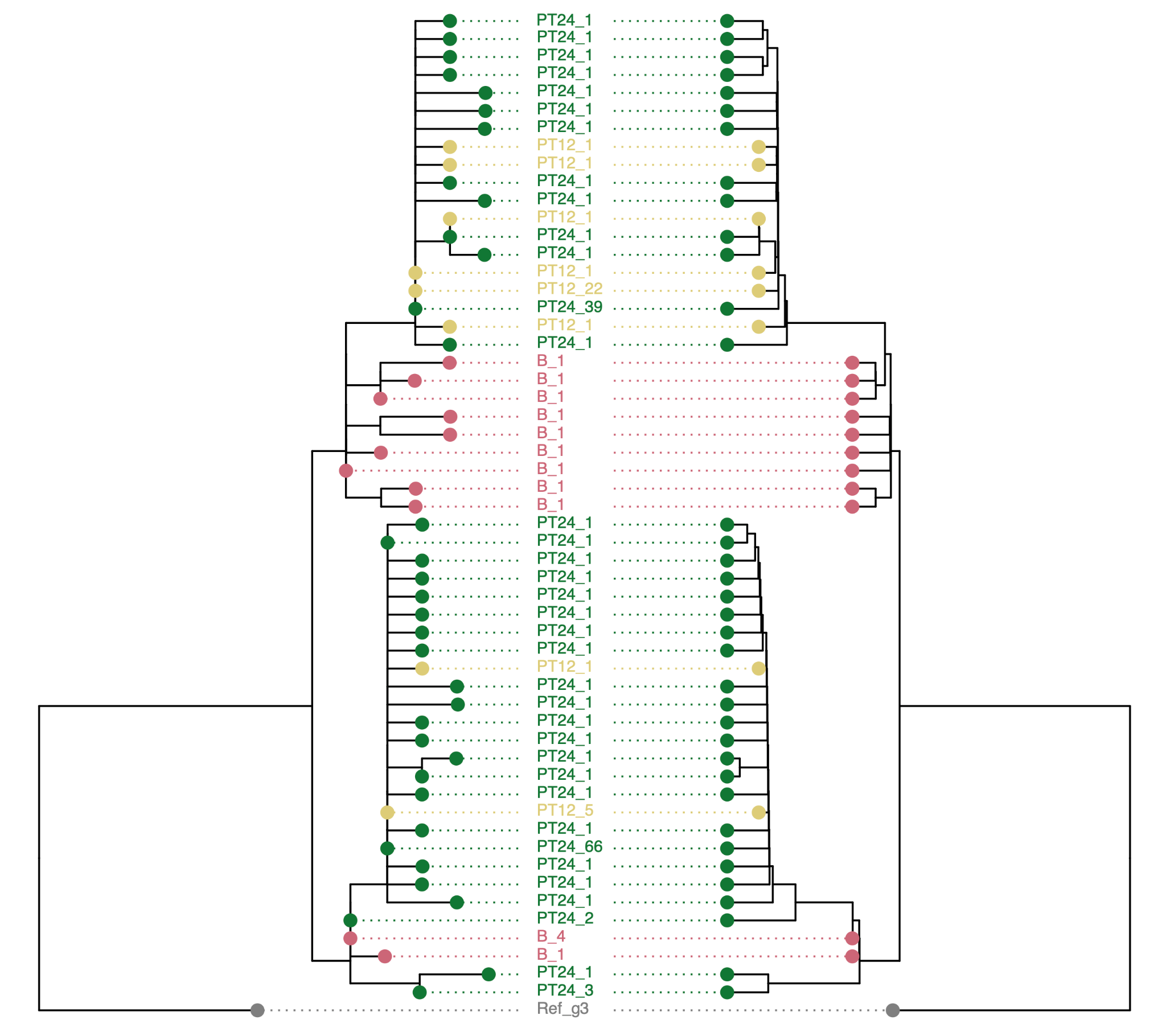

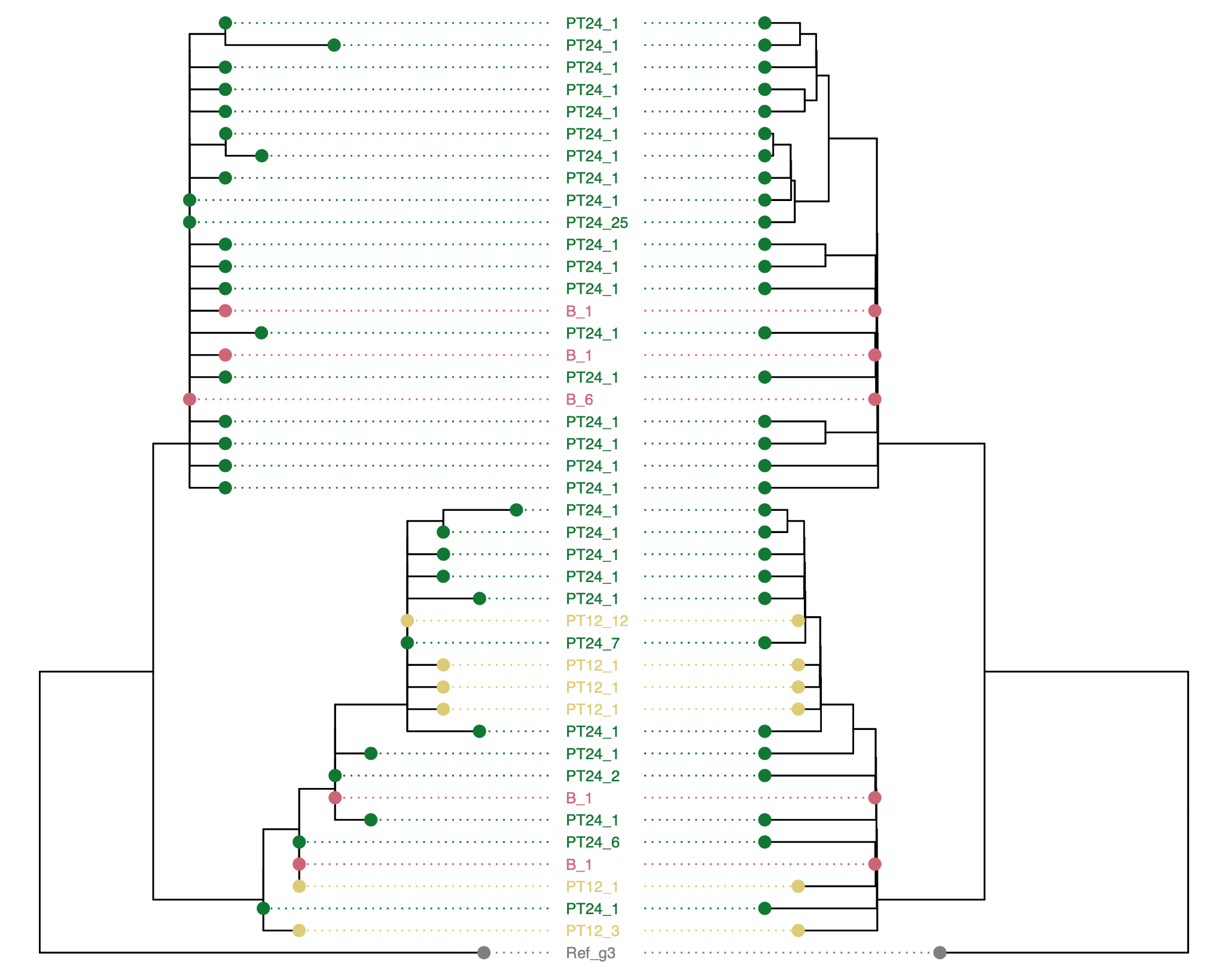

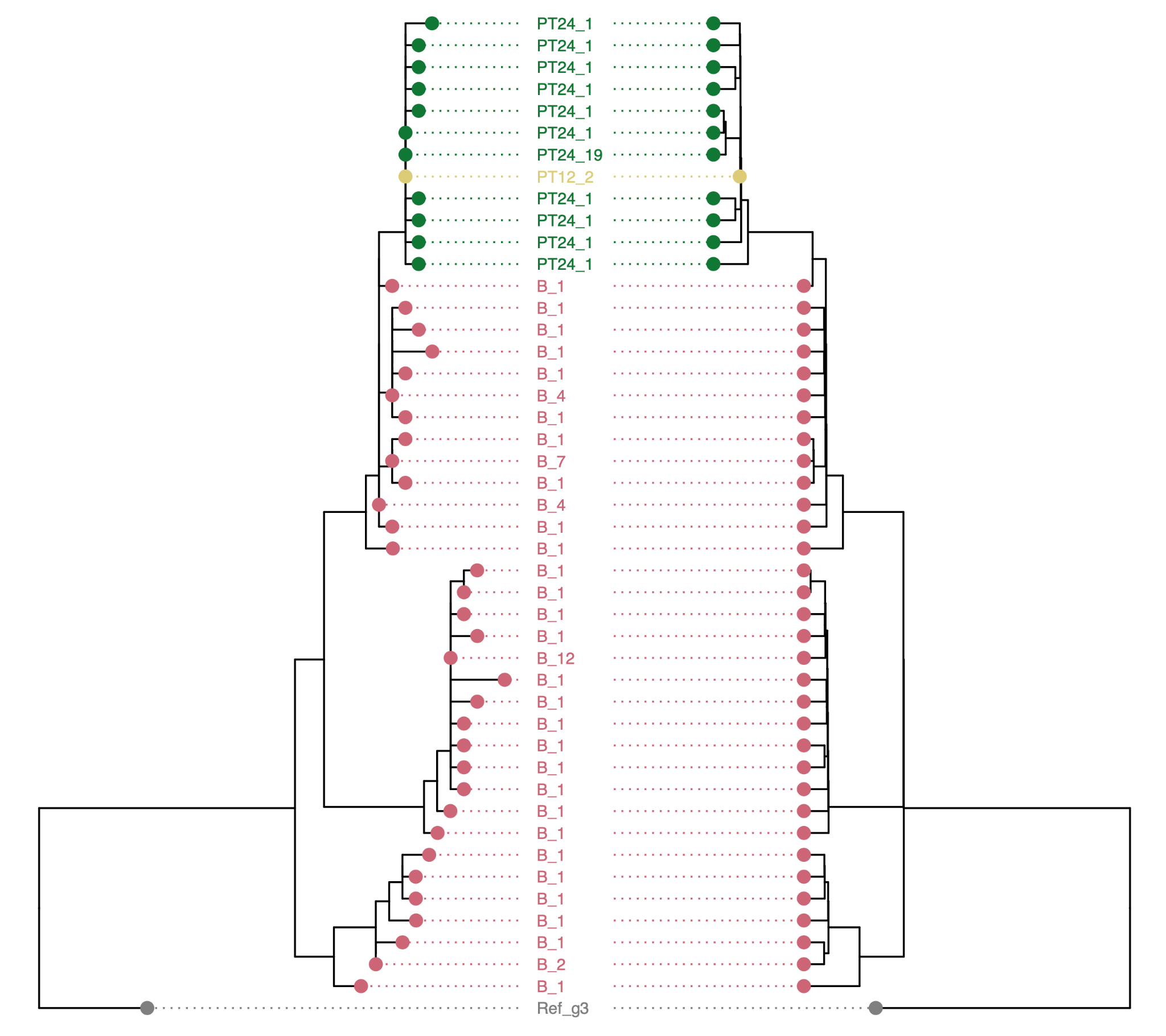

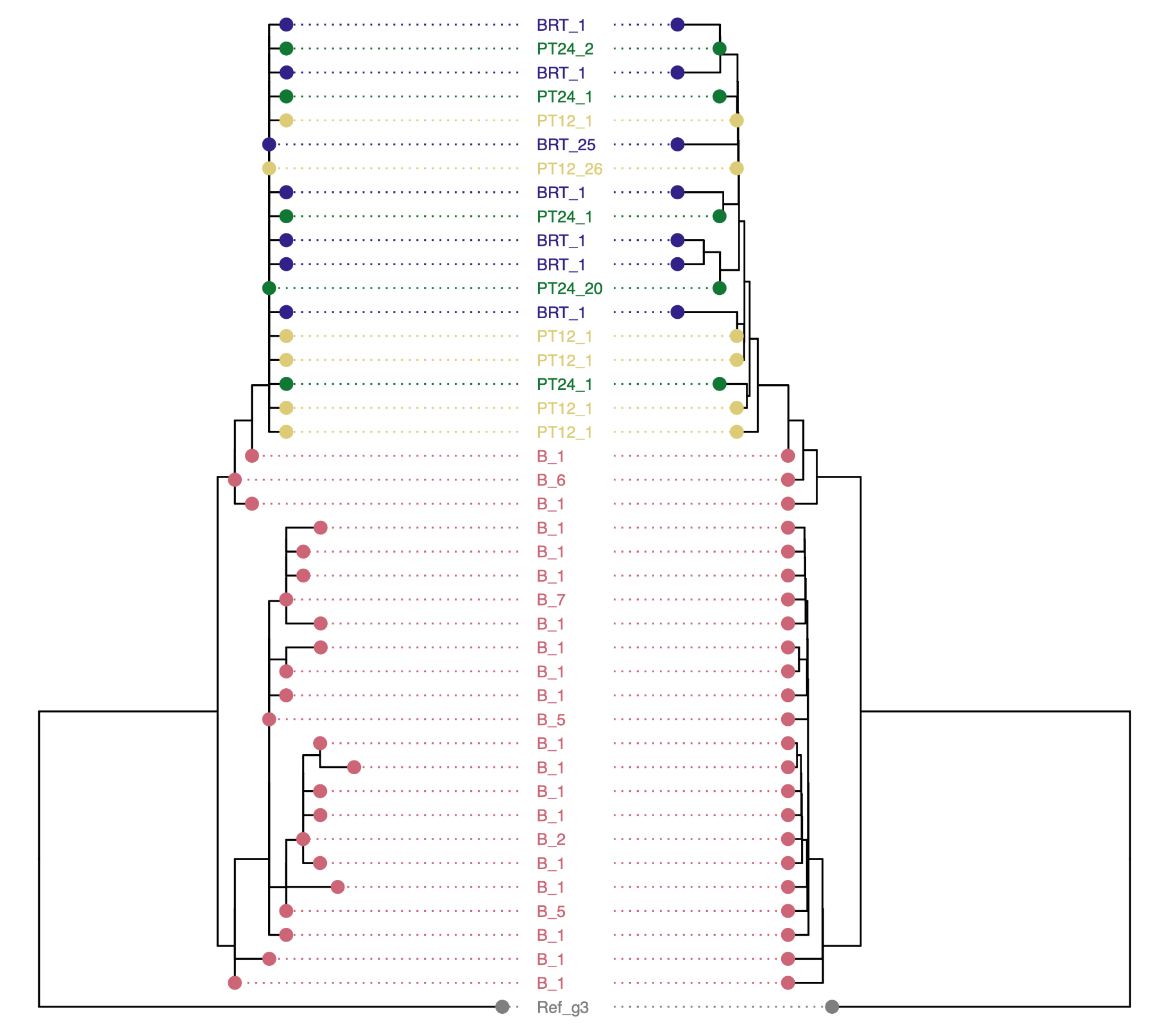

Supplement: S5 Fig — The top two sets of phylogenies show structure maintenance while the bottom two sets do not show clear structure maintenance of the within-host viral population. The left side of each set is the molecular phylogeny, where branch length corresponds to the number of substitutions. The right side of each set is the time-calibrated phylogeny, where branch length corresponds to sampling time. The tip labels indicate the sampling time point and the number of occurrences the sequence appeared within the window. Red: Baseline (B), yellow: 12 weeks post treatment (PT12), green: 24 weeks post treatment (PT24), and blue: baseline before retreatment (BRT). (DOCX) [file ppat.1012959.s005.docx]

**
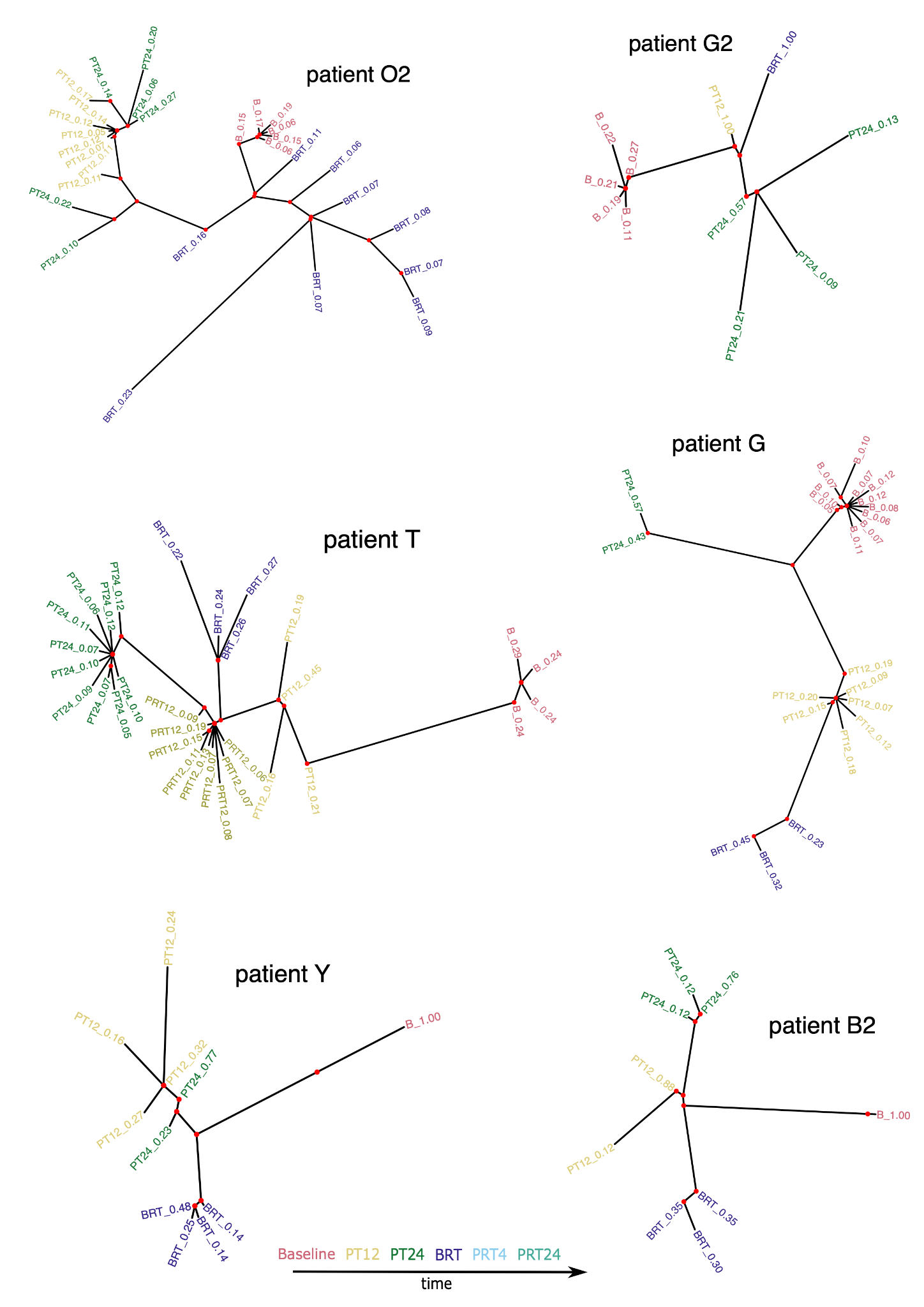
**

Supplement: S6 Fig — Baseline sequences are in red, PT12 (post treatment 12 weeks) sequences are in yellow, PT24 (post treatment 24 weeks) sequences are in green, BRT (baseline before re-treatment) sequences are in purple, PRT4 (post re-treatment 4 weeks) sequences are in light blue, PRT24 (post re-treatment 24 weeks) sequences are in light green. The tip labels consisted of the sampling time point and the CliqueSNV-estimated frequency of the haplotype within the sample. Note the haplotype frequencies from the same sample might not sum up to 1 as the minimum frequency to call a haplotype was set to 0.05. (DOCX) [file ppat.1012959.s006.docx]

**
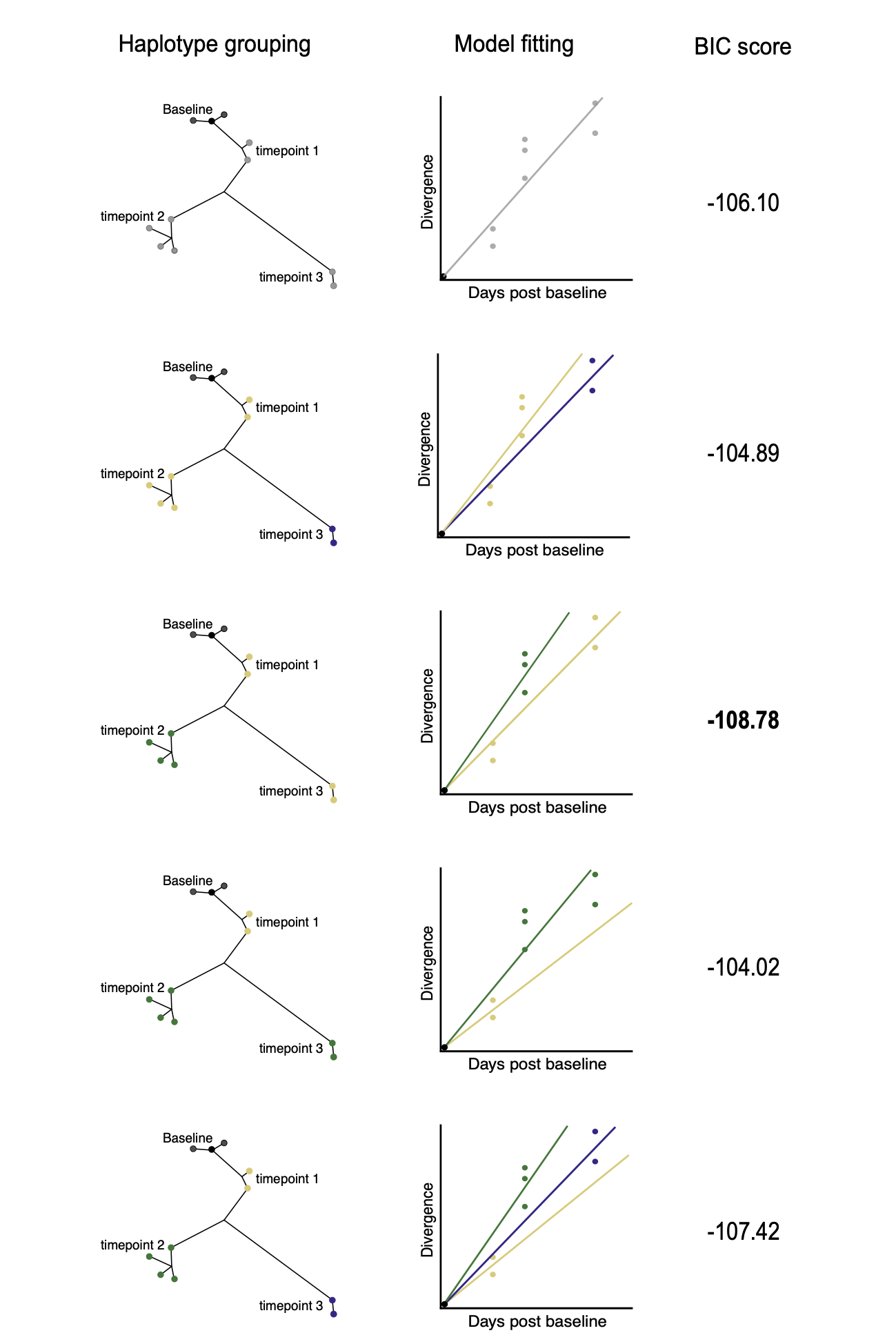
**

Supplement: S7 Fig — The first column lists all combinations of post Baseline haplotype grouping, as indicated by coloured tip labels. The second column shows the fitting of the linear mixed-effect models with the corresponding groupings and the third column shows the BIC score of the model fit. The bolded BIC score of -108.78 was the lowest among all models tested and suggested that for this individual, time point 1 and 3 haplotypes belong to the same lineage, while time point 2 haplotypes belong to a different lineage. (DOCX) [file ppat.1012959.s007.docx]
