# Supplementary material for: Genetically distinct within-host subpopulations of hepatitis C virus persist after Direct-Acting Antiviral treatment failure
Source: PLoS Pathog. 2025 Apr 1;21(4):e1012959. doi: 10.1371/journal.ppat.1012959 (PMC11981120; doi:10.1371/journal.ppat.1012959)
Supplement: S1 Text — (DOCX) [file ppat.1012959.s008.docx]

**S1 Text**

**Additional method specifications**

*Method for k parameter optimisation*

The *k* parameter in *phyloscanner* is used to identify collections of reads taken from the same sample which are too diverse to be the descendants of a single transmission event. This is used both to identify probable contaminant reads and to identify multiple infections. For brevity, we say this is a question of whether we are dealing with a *single event clade* or a *multiple clade event.* It works by introducing a penalty to the maximum parsimony reconstruction whereby a clade whose basal branches are sufficiently long will be reconstructed as being the result of two or more introductions to a host. When *k*=0, the reconstruction is simple maximum parsimony; increasing values of it decrease the amount of divergence along the basal branches at which the tips are split into two or more groups representing separate infection events. See the supplementary materials to Wymant et al. 2018 for full details [1].

The choice of a suitable *k* is not a straightforward one, particular given that *phyloscanner* is often run on hundreds of genomic windows for large numbers of samples. Branch lengths across the genome are often normalised when the package is used so that divergence between samples in different windows is on roughly the same scale. But this often leads to the branch lengths themselves being in largely arbitrary units. Even were it not so, the question of how much within-host diversity is too great to plausibly represent the result of a single infection event for any particular pathogen is a far from straightforward one. Here we outline one procedure to identify a suitable single value of the parameter for a large dataset consisting of sequences from a diverse collection of hosts.

The following procedure is intended to produce a single *k* that can be used on an entire dataset. It can be applied to the entire dataset, or if that is too large to be computationally feasible, a subset of it. The subset should be chosen to be genetically representative.

The assumption we make is that the full dataset is sufficiently diverse that if two sequences from the same host do not represent a single introduction to that individual, then they do not form a monophyletic clade in a phylogeny of all samples; the dataset’s background diversity is such that this does not happen. For small numbers of samples this may not be true, but adding sequences to make it more plausible need not be a matter of analysing more BAMs of short reads - simply adding a diverse collection of consensus sequences to the analysis will have the same effect.

In a bifurcating phylogeny, the statement that a set of *n* tips form a monophyletic clade is equivalent to the statement that the clade descended from their MRCA has 2*n*-1 internal nodes. This is also true if we are not considering tips, but the root nodes of *n* non-overlapping subclades with all child nodes pruned (such that they become tips in a subtree). If we assume that a clade is monophyletic if and only if the tips from it represent a single infection, and then this means that a set form a single event clade if and only if, for every collection of *n* nodes in the subtree descended from the MRCA of those tips, with the nodes of subtrees rooted at each of the *n* being non-overlapping, there are 2*n*-1 distinct nodes on the paths joining each of those *n* subtree MRCAs to the overall MRCA (including the overall MRCA itself).

This property can thus be used as a gold standard for testing if a clade is a single event clade. We now want to find the best value of *k* as a classifier. We can do this by, for each sample in each *phyloscanner* window (with normalised branches):

1. Prune the phylogeny so only tips from that sample remain.
2. Set *k*=0
3. Run the *phyloscanner* parsimony algorithm to split the tips into *n* groups
4. If *g*>1, record *k’=k* and the *n* MRCA nodes of the *g* groups and stop
5. If not, increase *k* by a fixed, small amount *i* (we used *i*=0.1) and go to step 3
6. Check whether there are 2*n-*1 unique nodes on the paths from the *n* group MRCA nodes to the overall MRCA node of the sample in the unpruned tree.

If there are 2*n*-1 such nodes then the sample forms a single event clade in this window according to our gold standard; if there are more than 2*n*-1 then it does not. In the former case, using values of *k* of at least *k’* result in a false positive identification of a multiple clade event, and values less than or equal to *k’-i* are true negatives. In the latter case, values of at least *k’* are true positives and values less than or equal to *k*’-*i* are false negatives. This can be used to estimate sensitivity and specificity for *k* equal to any multiple of *i*, and an optimal value for it using a ROC curve or any other means of optimising the value of a binary classifier. See figure 1 for an illustration.


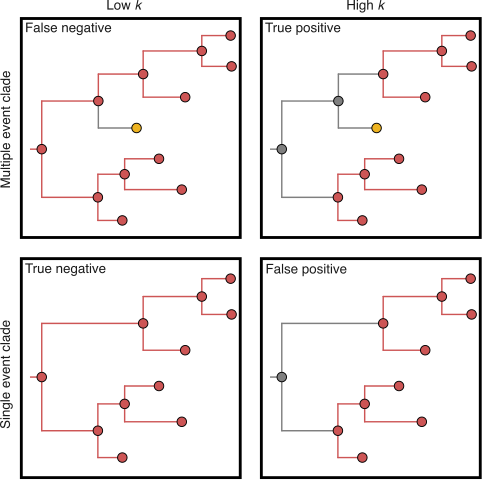


**Figure 1:** Use of *k* as a binary classifier for a phylogeny representing a multiple clade event. All red tips are derived from the same host; the yellow tip comes from a different host. It is assumed that the presence of the yellow tip indicates that this is not a single event clade, and vice versa. A *k* that is too low will miss this (top right). The yellow tip is missing from the bottom row, but a *k* that is too large (bottom right) will nevertheless declare a multiple clade event.

A naive version of this gold standard has a limitation where tips from another host are nested within the clade of interest because of precedence of the latter in the chain of transmission. In figure 1 this would happen if the host represented by the yellow tip was infected by the one represented by the red, possibly by way of one or more unsampled individuals. To refine the gold standard condition, we also require that, for a sample to be identified as the result of a multiple clade event, the longest branch within the subclades of the tips associated with each split is shorter than the mean of the patristic distances between the MRCAs of those splits. See figure 2 for an example.


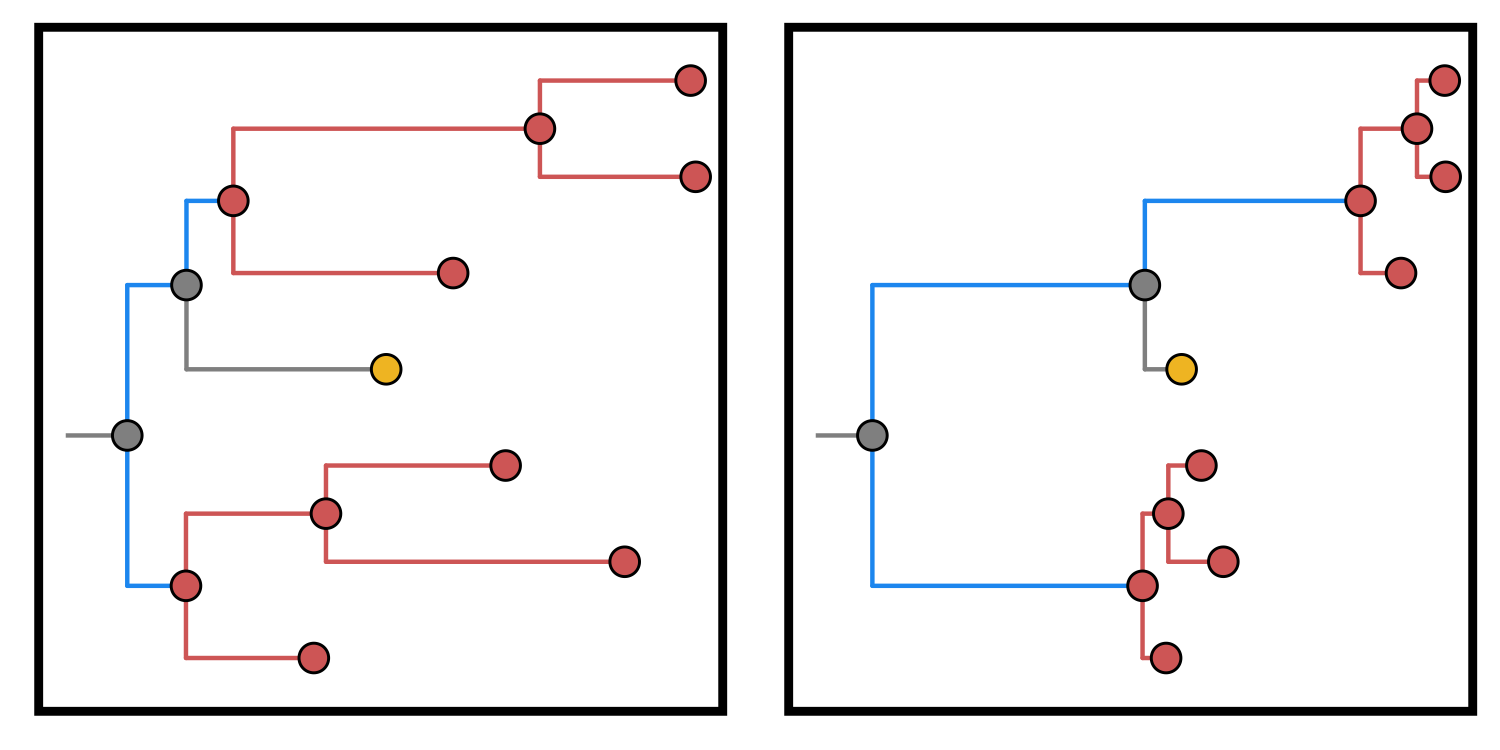


**Figure 2:** On the left, the appearance of the yellow tip nested in the diversity of the red sample is more likely to indicate the gold sample is descended from that diversity than multiple introductions to the red host occurred. The naive version of the gold standard would classify this as a multiple clade event, just as it would the right-hand tree whose basal branch lengths very much do suggest a multiple clade event. To guard against this, we also stipulate that the call of a multiple clade event requires that the longest within-clade branch (longest red branch) is longer than the patristic distance between clade MRCAs (sum of blue branches). If there are more than two such clades (not shown), the mean patristic distance between them is used.

*phyloscanner_make_trees.py command for example window 101 to 310:*

python phyloscanner_make_trees.py path_to_bamfiles.csv -P -A HCV_references.fasta -2 H77_NC_038882 –min-read-count 1 --x-mafft mafft --x-iqtree iqtree2

--windows 100,309

*phyloscanner_analyse_trees.R command:*

Rscript phyloscanner_analyse_trees.R iqtreefiles_GTR+F+R6/iqtree_ BOSON_PTs s,24 -og H77_NC_038882 -m 1E-5 -od phyloscanner_output_pbk24 -x ^([0-9]+)[0-9_A-Z]+_read_([0-9]+)_count_([0-9]+)$ -rda -blr -ow -nr HCV_normalisation_g3a_By_Position.csv -db DuplicationData/DuplicateReadCountsProcessed_InWindow_ -tfe .treefile -pbk 24 -rwt 3 -rtt 0.01 -rcm -swt 0.5 -sdt 0.02 -amt -sat 0.33 -v 1

*cliqueSNV command:*

java -jar clique-snv.jar -m snv-illumina -in file.bam -log -outDir cliqueSNV_output/ -tf 0.05 -fdf extended

**Reference**

Wymant, C., Hall, M., Ratmann, O., Bonsall, D., Golubchik, T., de Cesare, M., et al. PHYLOSCANNER: inferring transmission from within-and between-host pathogen genetic diversity. *Molecular biology and evolution*, 2018; *35*(3), 719-733.
