# Supplementary material for: Genetically distinct within-host subpopulations of hepatitis C virus persist after Direct-Acting Antiviral treatment failure
Source: PLoS Pathog. 2025 Apr 1;21(4):e1012959. doi: 10.1371/journal.ppat.1012959 (PMC11981120; doi:10.1371/journal.ppat.1012959)
Supplement: S1 Table — (DOCX) [file ppat.1012959.s009.docx]

**S1 Table** List of 65 resistance-associated variants (RAVs).

| **Gene** | **Site** | **RAV** | **Relevant DAA** | **Publication** |
| --- | --- | --- | --- | --- |
| NS5A | 28 | T | Daclatasvir | [1] |
| NS5A | 30 | K | Daclatasvir | [1] |
| NS5A | 31 | M | Daclatasvir | [1] |
| NS5A | 32 | L | Daclatasvir | [1] |
| NS5A | 54 | H | Daclatasvir | [1] |
| NS5A | 58 | S | Daclatasvir | [1] |
| NS5A | 62 | P | Daclatasvir | [2] |
| NS5A | 92 | K | Daclatasvir | [1] |
| NS5A | 93 | N | Daclatasvir | [1] |
| NS5A | 93 | H | Daclatasvir | [1] |
| NS5A | 172 | E | Daclatasvir | [2] |
| NS5A | 176 | V | Daclatasvir | [2] |
| NS5A | 276 | T | Daclatasvir | [2] |
| NS5A | 294 | T | Daclatasvir | [3] |
| NS5A | 442 | S | Daclatasvir | [3] |
| NS5B | 66 | T | Sofosbuvir | [2] |
| NS5B | 90 | A | Sofosbuvir | [2] |
| NS5B | 117 | N | Sofosbuvir | [2] |
| NS5B | 120 | R | Sofosbuvir | [2] |
| NS5B | 150 | V | Sofosbuvir | [4] |
| NS5B | 159 | F | Sofosbuvir | [1] |
| NS5B | 180 | Q | Sofosbuvir | [2] |
| NS5B | 185 | G | Sofosbuvir | [2] |
| NS5B | 282 | T | Sofosbuvir | [1] |
| NS5B | 293 | I | Sofosbuvir | [2] |
| NS5B | 316 | H | Sofosbuvir | [5] |
| NS5B | 316 | N | Sofosbuvir | [5] |
| NS5B | 320 | F | Sofosbuvir | [5] |
| NS5B | 321 | A | Sofosbuvir | [1] |
| NS5B | 401 | R | Sofosbuvir | [2] |
| NS5B | 517 | K | Sofosbuvir | [3] |
| NS5B | 571 | Y | Sofosbuvir | [3] |
| NS2 | 96 | S |  | [2] |
| NS2 | 119 | A |  | [5] |
| NS2 | 131 | S |  | [3] |
| NS2 | 132 | I |  | [5] |
| NS2 | 132 | V |  | [5] |
| NS3 | 67 | V |  | [5] |
| NS3 | 244 | R |  | [2] |
| NS3 | 256 | T |  | [2] |
| NS3 | 264 | R |  | [2] |
| NS3 | 315 | V |  | [2] |
| NS3 | 318 | T |  | [2] |
| NS3 | 354 | L |  | [2] |
| NS3 | 390 | E |  | [3] |
| NS3 | 418 | F |  | [2] |
| NS3 | 426 | I |  | [2] |
| NS3 | 559 | Y |  | [2] |
| N53 | 609 | T |  | [2] |
| NS3 | 620 | T |  | [2] |
| E2 | 61 | Y |  | [2] |
| E2 | 118 | N |  | [3] |
| E2 | 138 | A |  | [3] |
| E2 | 178 | L |  | [2] |
| E2 | 193 | E |  | [3] |
| C | 42 | P |  | [2] |
| C | 60 | E |  | [3] |
| C | 109 | P |  | [3] |
| C | 109 | S |  | [2] |
| E1 | 157 | I |  | [2] |
| E1 | 181 | T |  | [3] |
| E1 | 181 | I |  | [2] |
| NS4B | 48 | A |  | [2] |
| NS4B | 114 | S |  | [3] |
| NS4B | 162 | F |  | [2] |

Reference

1. Smith D, Magri A, Bonsall D, Ip CLC, Trebes A, Brown A, et al. Resistance analysis of genotype 3 hepatitis C virus indicates subtypes inherently resistant to nonstructural protein 5A inhibitors. Hepatology. 2019;69: 1861–1872.
2. Ansari MA, Pedergnana V, L C Ip C, Magri A, Von Delft A, Bonsall D, et al. Genome-to-genome analysis highlights the effect of the human innate and adaptive immune systems on the hepatitis C virus. Nat Genet. 2017;49: 666–673.
3. Ansari MA, Aranday-Cortes E, Ip CL, da Silva Filipe A, Lau SH, Bamford C, et al. Interferon lambda 4 impacts the genetic diversity of hepatitis C virus. Elife. 2019;8. doi:[10.7554/eLife.42463](http://dx.doi.org/10.7554/eLife.42463)
4. Wing PAC, Jones M, Cheung M, DaSilva S, Bamford C, Jason Lee W-Y, et al. Amino Acid Substitutions in Genotype 3a Hepatitis C Virus Polymerase Protein Affect Responses to Sofosbuvir. Gastroenterology. 2019;157: 692–704.e9.
5. Smith DA, Fernandez-Antunez C, Magri A, Bowden R, Chaturvedi N, Fellay J, et al. Viral genome wide association study identifies novel hepatitis C virus polymorphisms associated with sofosbuvir treatment failure. Nat Commun. 2021;12: 6105.
